# Lymphocytes are less sensitive to autophagy than monocytes during fasting and exercise conditions

**DOI:** 10.1007/s10495-022-01752-x

**Published:** 2022-07-19

**Authors:** Julia M. Kröpfl, Christian Morandi, Benedikt A. Gasser, Raphael Schoch, Arno Schmidt-Trucksäss, Marijke Brink

**Affiliations:** 1 Department of Sport, Exercise, and Health, Sport and Exercise Medicine, University of Basel, Grosse Allee 6, 4052 Basel, Switzerland; 2 Department of Biomedicine, University Hospital Basel and University of Basel, Hebelstrasse 20, 4031 Basel, Switzerland

**Keywords:** Apoptosis, Autophagy, Fasting, Exercise, Mononuclear cells

## Abstract

The concomitant investigation of apoptosis (a regulated cell death) and autophagy (a conserved cell survival mechanism) in immune cells is rare. More detailed knowledge of these two types of self-consumption in circulating lymphocytes and monocytes would be important, since conditions such as fasting and acute exercise could promote health by a coordinated/linked modulation of autophagy and apoptosis in these mononuclear cells. In this study we performed flow cytometry to quantify numbers of apoptotic and autophagic mononuclear cells, lymphocytes and monocytes in fasting, standardized fed, and exercise conditions, using Annexin V, LC3B, and p62, respectively. We show that within total mononuclear cells lymphocytes are less apoptotic and autophagic than monocytes during fasting (p < 0.001, p < 0.05, respectively) and after acute exercise (p < 0.01, p < 0.05, respectively). Fasting increased circulating autophagic monocyte concentrations, but not lymphocytes compared to the fed control condition. Acute exercise elevated circulating autophagic lymphocyte concentrations, but not monocytes. Interestingly, Western blotting analysis of the fasting samples showed that higher LC3BII/I ratios were correlated with lower numbers of autophagic mononuclear cells (r = − 0.74, p = 0.02, n = 8), which could be attributed to the monocyte subgroup, but not lymphocytes. These results extend the current knowledge of the two types of self-consumption in circulating immune cells and underline their possible importance in pro-inflammatory monocytes during fasting and exercise as health promoting interventions.

## Introduction

For a unicellular organism, cell death is terminal. For a multicellular organism, cell death is an important biological process for physiological development and cellular growth [1]. Deregulation of cell death leads to the pathogenesis of multiple human diseases [1]. Apoptosis and autophagy are two cell death/survival mechanisms that run in parallel within a cell and are considered types of self-consumption [2]. Intrinsic apoptosis—a regulated cell death—is characterized in its early and mid-phase by the binding and inactivation of pro-survival B cell lymphoma (Bcl) family member Bcl-2 and the subsequent exposure of phosphatidylserine (PS)—an “eat-me” signal—to the extracellular side of the cell membrane, respectively [3]. On the contrary, autophagy is considered a conserved survival mechanism, which is accompanied by the formation of the autophagosome, a bilayer vesicle containing damaged organelles, proteins, and other cytoplasmic components [4]. The autophagosomes fuse with the lysosomes, which are responsible for degrading cellular macromolecules and organelles and producing renewable energy and metabolites [4]. Microtubule-associated protein 1A/1B-light chain 3 (LC3) is commonly used to detect autophagosomes [5] and LC3BII was recently shown to be one of the most reliable markers to measure autophagy in human blood [6]. Also, autophagic cargo marked with Lys63‑linked ubiquitin chains for uptake by autophagosomes can interact with a series of adaptors such as sequestosome 1 (SQSTM1, p62), which is destroyed during autophagic flux [2]. Autophagy can be described as a degradation mechanism rather than a form of cell death, although it might also induce cell death in special (patho)physiological conditions [7, 8]. Intrinsic apoptosis and autophagy may be induced simultaneously or successively when cells are exposed to certain stimuli [1]. Autophagy often serves as a cytoprotective mechanism and is induced when cells are exposed to low-intensity stress, whereas apoptosis is initiated if autophagy is inhibited or ineffective at higher stress-intensities [1, 2]. In a previous study we could show that lymphocytes and monocytes were differently affected by cell apoptosis simply induced by mechanical stress from the cell isolation [9]. Interestingly, we found that lymphocytes were almost unaffected while monocytes showed a higher apoptosis-induction measured by flow cytometry detection of PS-bound Annexin-V [9].

To further investigate the underlying reasons—since the extent of autophagy acts as a regulator of apoptosis, also within a homogenous cell population [10]—we aimed to 1. elucidate if lymphocytes and monocytes would be differently affected by both forms of self-consumption, and 2. validate the simultaneous flow cytometric measurement of apoptosis and autophagy by Western blot.

## Methods

### Study design and methodological approach

Peripheral blood of healthy human participants was investigated (n = 8). 18 ml of blood were withdrawn from the cubital vein into tubes coated with EDTA.

Apoptosis and autophagy were induced by two different conditions: (A) by fasting for 14–15 h, the last meal was consumed at 9 pm the day before. Since coffee enhances apoptosis [11] and autophagy [12] induction, participants were allowed to consume up to two cups of coffee without milk or sugar in the morning prior to blood sampling. The blood sample was withdrawn between 11am and 12pm (fasting condition); (B) by an exhaustive acute incremental exercise test (exercise condition) on a bicycle ergometer (Sport Excalibur, Lode Medical Technology, Groningen, The Netherlands), as it was also shown to induce both apoptosis [13] and autophagy [14]. The ramp protocol was selected based on participants’ fitness status (protocol 4: warm-up 50 Watt, then increase of 20 W/min; protocol 5: warm-up 50 Watt, then increase of 30 W/min). As a control to the fasting and exercise condition, participants had a standardized breakfast in the morning of the exercise test (2 croissants, no coffee, water ad libitum) and an identical power bar before the acute exercise (fed condition), which was also performed between 11am and 12pm. Directly before (fed condition) and up to 5′ after the acute exercise (exercise condition), two additional blood samples were collected. Fasting and fed/exercise conditions were taken at least one week apart.

### Cell isolation and flow cytometry

**Fig. 1:**
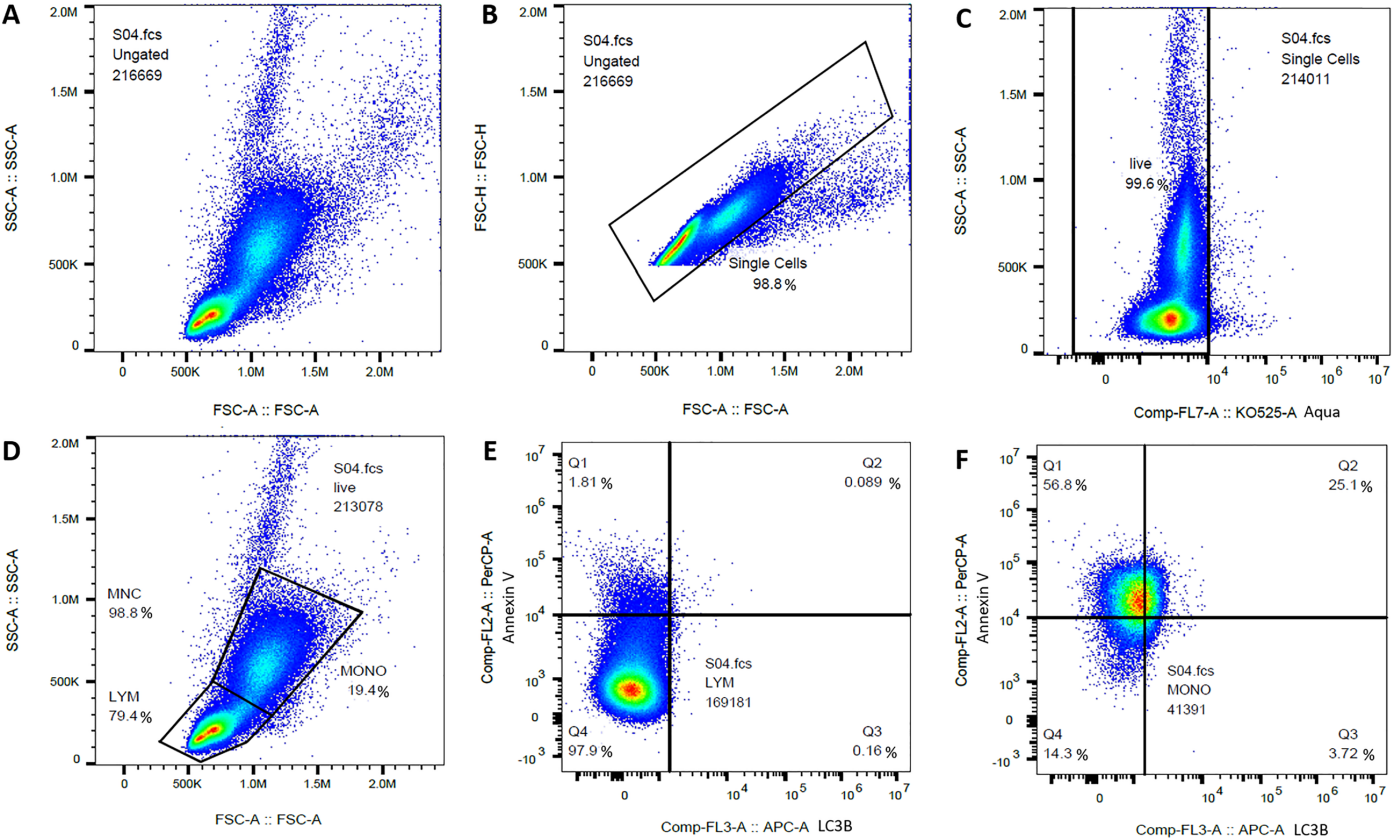
Flow cytometry gating strategy. Total acquired mononuclear cells (MNC) (**A**) were gated for singlets (**B**) and live cells (**C**). Then, total MNC were divided into lymphocyte (LYM) and monocyte (MONO) subgroups (**D**) and these were separately (LYM: **E**, MONO: **F**) as well as together analyzed for apoptosis (Annexin V) and autophagy (LC3B)

Mononuclear cells were isolated by density gradient centrifugation within 2 h of blood withdrawal and cells were re-suspended in 2 ml PBS before flow cytometry staining. Staining was based on the protocol by Bergamaschi and colleagues [15] and our own work [9]. All samples were measured in duplicate. In brief, more than two million mononuclear cells were incubated with stain buffer (FBS, BD Biosciences, Allschwil, Switzerland) for 5 min before being stained with a live/dead marker (fixable Aqua dead cell stain kit, Thermofisher Scientific, Reinach, Switzerland) and an apoptosis marker (Annexin V-PerCP-Cy 5.5, BD Biosciences, Allschwil, Switzerland) at room temperature for 10 min. After washing, mononuclear cells were permeabilized and fixed (Fixation/Permeablization Kit, BD Biosciences, Allschwil, Switzerland, 5°C, 20 min) according to the manufacturer’s instructions. After another washing step, mononuclear cells were stained with an intra-cellular marker for autophagy for 30 min (LC3B Alexa Fluor 647, Bio-Techne AG, Zug, Switzerland). After a last wash, cells were analyzed in stain buffer on a Cytoflex device. Cell analysis was done with FlowJo (Version 10.6.2, BD Biosciences, Allschwil, Sitzerland). At least 200,000 mononuclear cells (Fig. 1A) were gated. After excluding doublets (Fig. 1B) and debris (Fig. 1C) by using the live/dead marker (Aqua), live lymphocytes and monocytes were separately as well as together (MNC, Fig. 1D) investigated for their amount of apoptosis and autophagy (Fig. 1E, F). Apoptotic and autophagic cell numbers were calculated—considering respective fluorescence-minus-one (FMO) background fluorescence—by multiplying total percentages of the parent population (mononuclear cells, lymphocytes or monocytes) for either Annexin V^+^ or LC3B^+^ cells by circulating absolute lymphocyte and/or monocyte counts and are given in cells/µl. All cell concentrations after exercise were corrected for plasma changes due to dehydration [16].

### Western blot

**Fig. 2:**
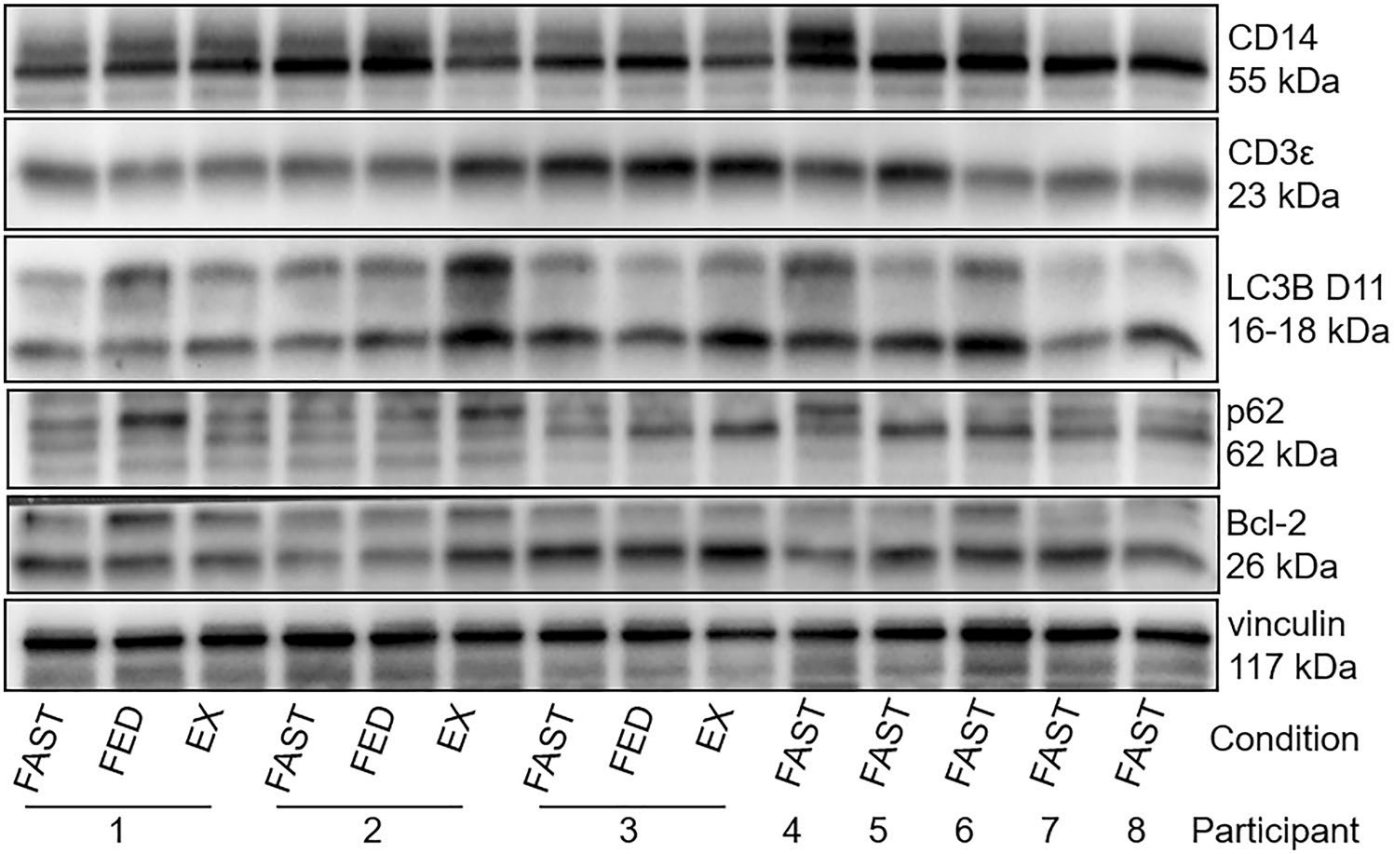
Representative Western blotting analyses. Protein extracts of mononuclear cells were stained for the monocyte marker CD14, the lymphocyte marker CD3ε, the autophagy markers LC3B D11 and SQSTM1 (p62), the apoptosis marker Bcl-2 and vinculin as loading control. *FAST* fasting condition, *EX* exercise condition

To validate the flow cytometry results, Western blotting analyses were performed [17] with total protein extracts of mononuclear cells, using antibodies against Bcl-2 (Santa Cruz Biotechnology, Texas, USA), LC3B D11 (BioConcept, Allschwil, Switzerland), and SQSTM1 (p62, Abcam, Cambridge, UK). A higher LC3BII/I ratio and a lower protein expression of p62 may indicate increased autophagy. For p62, the band running at 62 kDa as well as the higher bands that may reflect its posttranslational modification, were analyzed individually and together. To compare the relative amounts of lymphocyte or monocyte protein with their numbers found by flow cytometry, Western blots were incubated with antibodies to CD3ε and CD14 (both R&D Systems, Minneapolis, USA) for lymphocyte and monocyte detection, respectively. Vinculin (Santa Cruz Biotechnology, Texas, USA) was used as loading control. Please find the representative Western blot images in Fig. 2.

### Statistics

Data are given as individual values or mean ± standard deviation. Differences between conditions were calculated by repeated-measures ANOVA with post-hoc comparisons (LSD). Pearson or Spearman correlation analysis was done to investigate parameter associations. A p-value < 0.05 was considered significant.

## Results

### Whole blood cell counts

White blood cells (p < 0.001) as well as absolute lymphocytes (p < 0.001), monocytes (p < 0.001), neutrophils (p = 0.024), and mean corpuscular volume (p = 0.013) showed a main effect of condition, which came from an exercise-induced increase for all mentioned variables (Table 1).

**Table 1:** Blood cell counts

Blood collection	Fasting	Fed	EX
WBC, 10^9^/l	5.6 ± 0.8	5.5 ± 1.6	9.1 ± 1.7***^§§^
RBC, 10^12^/l	4.93 ± 0.46	4.86 ± 0.45	4.99 ± 0.49
Hct, %	43.4 ± 3.0	42.7 ± 3.4	45.7 ± 4.0
Hgb, g/l	151.6 ± 12.0	150.0 ± 14.4	157.0 ± 15.5
MCV, fl	88.3 ± 3.8	87.9 ± 3.8	89.8 ± 4.1*^§§^
Lymphocytes, 10^3^/µl	2.05 ± 0.56	1.90 ± 0.42	4.06 ± 0.88***^§§^
Monocytes, 10^3^/µl	0.45 ± 0.04	0.43 ± 0.16	0.69 ± 0.07***^§^
Neutrophils, 10^3^/µl	2.99 ± 0.87	3.11 ± 1.46	4.19 ± 1.76*^§^
Basophils, 10^3^/µl	0.02 ± 0.01	0.03 ± 0.01	0.05 ± 0.04

Values are reported as mean ± SD, n_Fasting_, _FED or EX_ = 5. Fasting, after fasting for 14-15 h; Fed, after a standardized breakfast (2 croissants, no coffee, water ad libitum) and an identical power bar right before the acute exercise; EX, 5’ after an exhaustive acute exercise test; WBC, white blood cell count; RBC, red blood cell count; Hct, hematocrit; Hgb, hemoglobin; MCV, mean corpuscular volume; For EX, cell concentrations are corrected for plasma changes due to dehydration; significant main effects of condition are indicated as follows: ***p < 0.001, *p < 0.05; to further specify these main effects, significant differences between the control condition (Fed) and Fasting or EX are indicated as follows: ^§§^p < 0.01, ^§^p < 0.05

### Analysis of apoptosis by flow cytometry

The percentage of *Annexin V*-positive mononuclear cells was comparable between conditions (Table 2), indicative of similar apoptosis induction. Calculation of the number of apoptotic mononuclear cells per µl blood also did not reveal any difference between the fasting (295 ± 64 cells/µl), fed (262 ± 109 cells/µl) and exercise (367 ± 99 cells/µl) conditions.

**Table 2:** Flow cytometry results for apoptosis and autophagy (as percent of the parent population)

Parameter	Fasting	Fed	EX
Annexin V + cells, %MNC	12.3 ± 3.5	12.4 ± 5.3	8.2 ± 4.7
Annexin V + cells, %LYM	1.6 ± 0.4	2.4 ± 1.4	1.7 ± 0.7
Annexin V + cells, %MONO	69.2 ± 9.2	63.1 ± 23.7	50.3 ± 17.9
LC3B + cells, %MNC	4.8. ± 3.2	3.1 ± 2.5	1.8 ± 1.3
LC3B + cells, %LYM	0.27 ± 0.13	0.13 ± 0.09	0.11 ± 0.03*
LC3B + cells, %MONO	26.4 ± 14.7	16.2 ± 11.8	11.8 ± 7.0*

Values are reported as mean ± SD, n_Fasting_, _Fed or EX_ = 5. Fasting, after fasting for 14–15 h; Fed, after a standardized breakfast (2 croissants, no coffee, water ad libitum) and an identical power bar right before the acute exercise; EX, 5’ after an exhaustive acute exercise test; MNC, mononuclear cells; LYM, lymphocytes; MONO, monocytes; significant main effects of condition are indicated as follows: *p < 0.05; post-hoc analysis did not indicate any significant differences between the control condition (Fed) and Fasting or EX

**Fig. 3:**
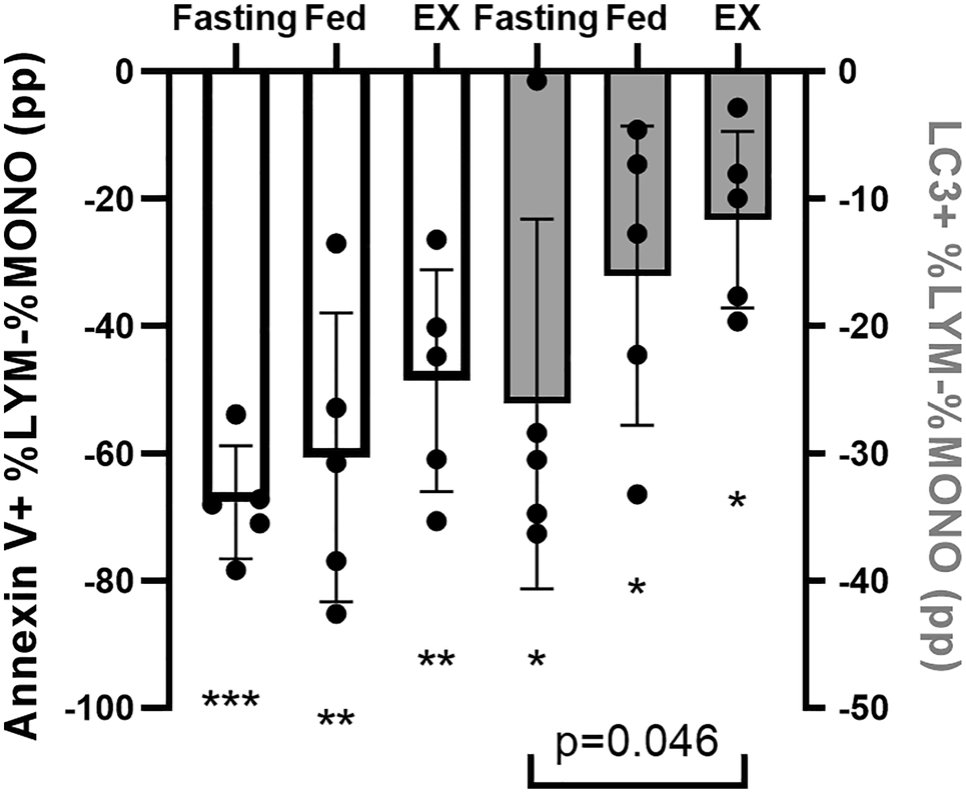
Differences (in percentage points, pp) between lymphocytes (LYM) and monocytes (MONO) regarding the amount of apoptosis and autophagy (given as %LYM or %MONO) during fasting, fed, and exercise (EX) conditions. MONO express on average significantly more of both types of self-consumption

**Fig. 4:**
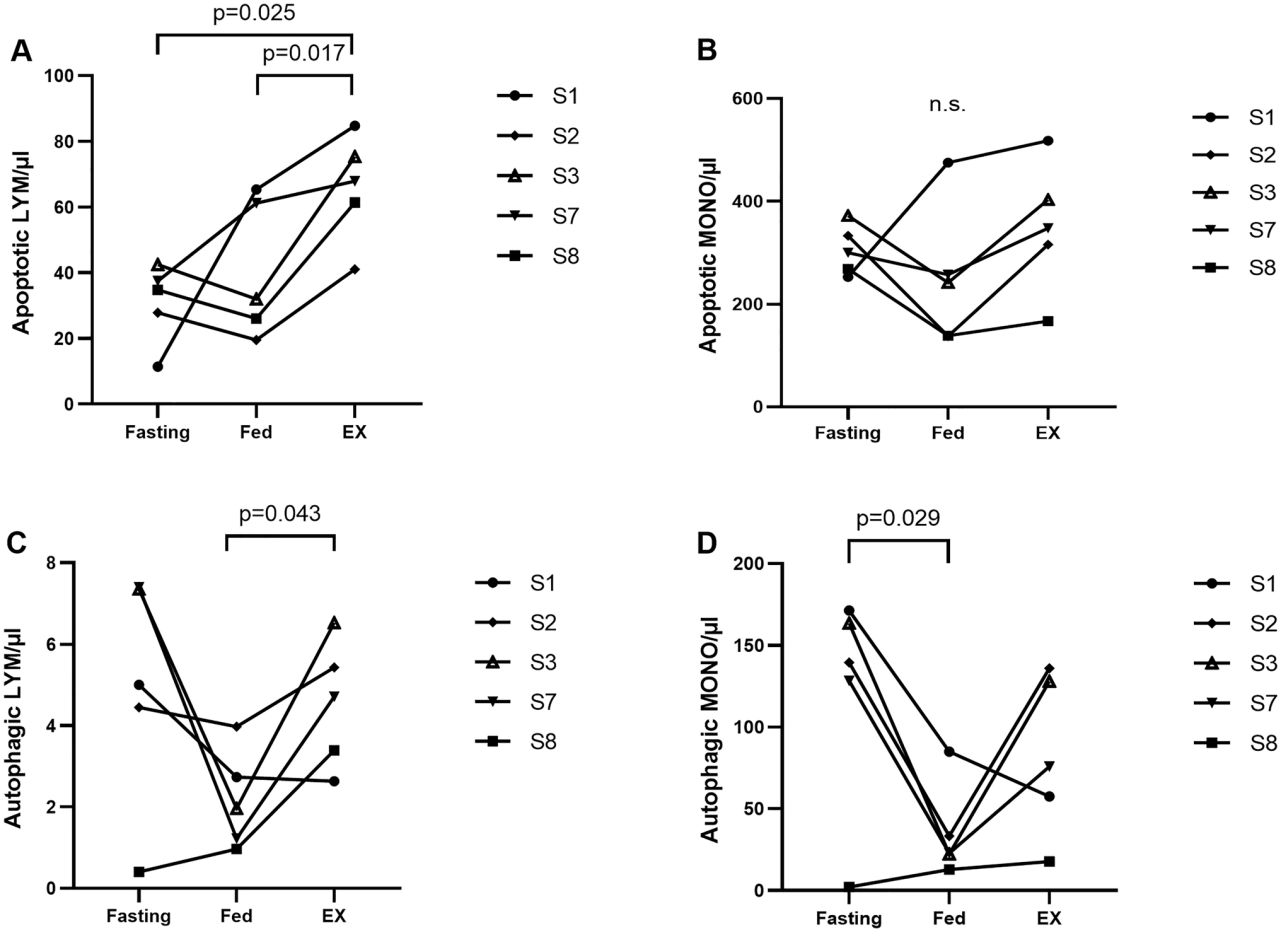
Flow cytometry results for apoptosis and autophagy (after back-calculation). Absolute circulating apoptotic or autophagic lymphocytes (LYM) and monocytes (MONO) expressed as cells/µl. Calculations were done by multiplying the percentage of each type of self-consumption per cell subgroup assessed by flow cytometry with circulating mature LYM or MONO concentrations assessed by a hematology analyzer. Displayed are apoptotic LYM/µl (**A**), apoptotic MONO/µl (**B**), autophagic LYM/µl (**C**), autophagic MONO/µl (**D**) during fasting, fed, and exercise (EX) conditions. Each participant (subject, S) is indicated by a solid line. p-values are indicated as exact numbers

As for the subgroups, the percentage of apoptotic lymphocytes was significantly lower than the percentage of apoptotic monocytes. The difference between the two mononuclear cell subgroups was − 67.7 ± 8.9 percentage points (pp, p < 0.001), − 60.7 ± 22.7 pp (p = 0.004) and − 48.6 ± 17.4 pp, (p = 0.003) for the fasting, fed and exercise condition, respectively (Fig. 3, white bars). Similar to what we found for the total mononuclear cell fraction, there were no significant differences between the conditions upon post-hoc analysis (Table 2, Fig. 3, white bars). However, there was a significant effect of condition for absolute numbers of apoptotic lymphocytes/µl (p = 0.021). They showed a significant increase from fed (41 ± 21 cells/µl) to exercise (66 ± 16 cells/µl, p = 0.017) and doubled from fasting (31 ± 12 cells/µl) to exercise (66 ± 16 cells/µl, p = 0.025, Fig. 4A). On the other hand, no significant differences in apoptotic monocytes/µl were detected between the fasting (305 ± 48 cells/µl), fed (250 ± 138 cells/µl) and exercise (350 ± 128 cells/µl) condition (Fig. 4B).

### Analysis of autophagy by flow cytometry

Labeling of LC3B was used to detect autophagic cells. The percentage of autophagic mononuclear cells was comparable between conditions (Table 2). Also when expressed/µl, autophagy in mononuclear cells was not different between fasting (112 ± 63 cells/µl), fed (80 ± 47 cells/µl) and exercise (85 ± 56 cells/µl).

In the lymphocyte subgroup, the percentage of autophagic cells was significantly lower than in the monocyte subgroup. The differences between lymphocytes and monocytes were − 26.1 ± 14.5 pp (p = 0.016), − 16.0 ± 11.8 pp, (p = 0.038), and − 11.7 ± 7.0 pp (p = 0.020) for the fasting, fed, and exercise condition, respectively (Fig. 3, grey bars). There was a significant difference between conditions (p = 0.049), being less pronounced after exercise compared to fasting (p = 0.046, Fig. 3, grey bars). Absolute numbers of autophagic lymphocytes/µl showed a statistical trend for condition (p = 0.071) resulting from significant increase from fed (2 ± 1 cells/µl) to exercise (5 ± 2 cells/µl, p = 0.043), while cell numbers stayed unchanged after fasting (5 ± 3 cells/µl), Fig. 4C. There was a significant effect of condition for autophagic monocytes/µl (p = 0.026). Numbers were significantly higher in the fasting (121 ± 69 cells/µl, p = 0.029) than in the fed (35 ± 29 cells/µl) condition, and stayed unchanged after exercise (83 ± 49 cells/µl), Fig. 4D.

### Analysis of apoptosis (Bcl-2) and autophagy (LC3B, p62) by Western blotting

Our Western blot analysis of Bcl-2, LC3BII/I and p62 in total mononuclear cells did not show any difference between fasting (Bcl-2: 1.07 ± 0.26; LC3BII/I: 1.14 ± 0.55; p62: 0.62 ± 0.06) or exercise (Bcl-2: 1.35 ± 0.50; LC3BII/I: 1.29 ± 0.62; p62: 1.15 ± 0.59) compared to the fed condition (set at 1).

### Correlation between apoptosis and autophagy by flow cytometry and validation by Western blot

**Fig. 5:**
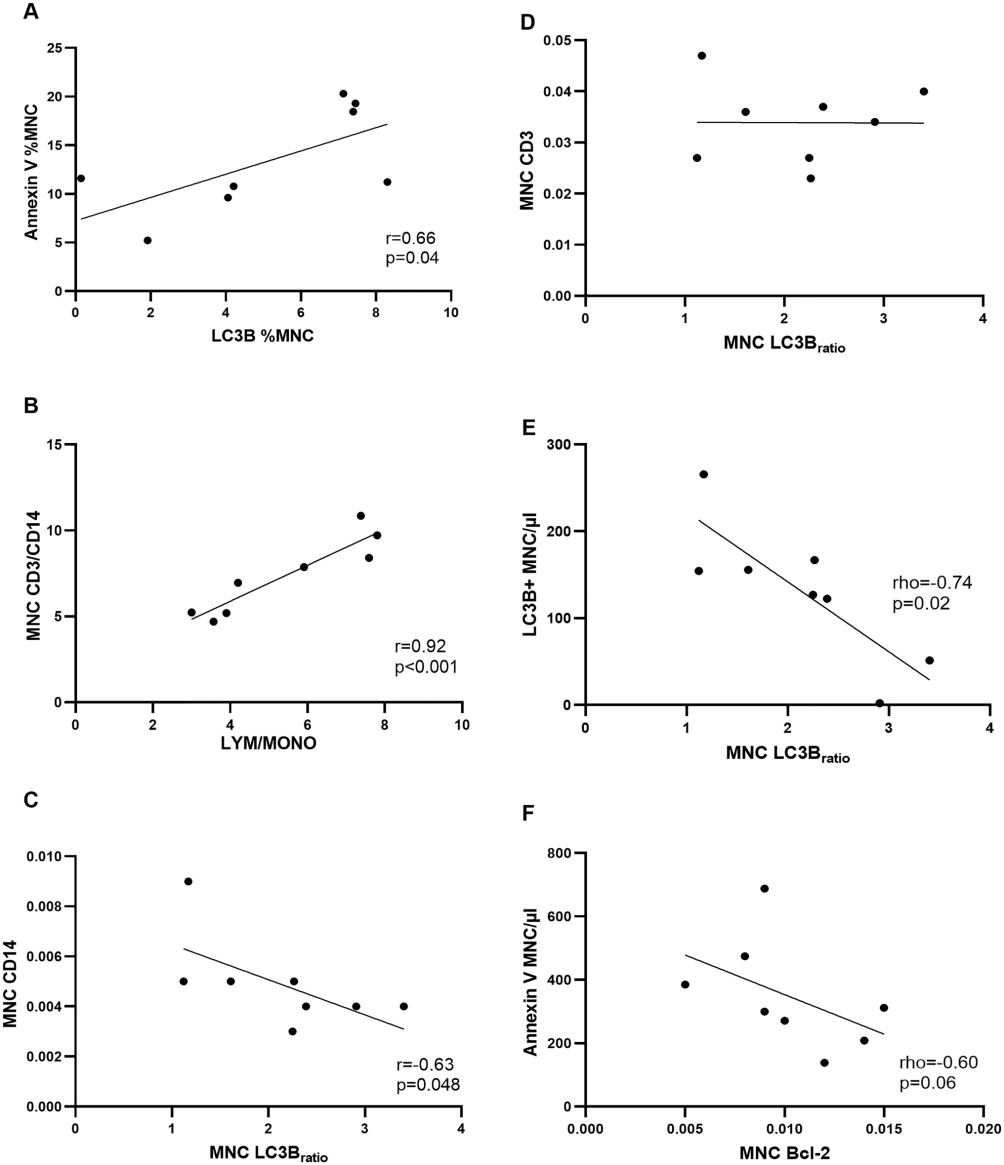
Apoptosis and autophagy correlations. The relative amount of apoptosis and autophagy in mononuclear cells (MNC) showed a significant positive relationship. When a higher percentage of MNC were apoptotic, also a higher percentage of cells were autophagic (**A**). The lymphocyte-to-monocyte (LYM/MONO) ratio from flow cytometry was significantly related to the CD3/CD14 ratio from Western blotting analysis (**B**). The amount of CD14 within the MNC protein extracts detected by Western blot was significantly indirectly related to the amount of MNC autophagy (**C**), whereas this association could not be found for CD3 (**D**). Circulating autophagic MNC/µl assessed by flow cytometry were significantly negatively related to the amount of LC3B_ratio_ (LC3BII/I) detected by Western blot (**E**). A trend for a similar association could be found between the number of circulating apoptotic MNC/µl assessed by flow cytometry and the amount of Bcl-2 detected by Western blot (**F**)

In the samples taken under fasting conditions, there was a significant positive association between the percentage of mononuclear cells positive for Annexin V and LC3B measured by flow cytometry (r = 0.66, p = 0.04, n = 8, Fig. 5A). LC3BII/I, but not p62, showed a statistical trend for a positive correlation (rho = 0.53, p = 0.09, n = 8) with Bcl-2 detected by Western blot. LC3BII/I and p62 were not associated (r = 0.43, p = 0.15, n = 8).

Subgroup analysis revealed that the lymphocyte-to-monocyte ratios (gated lymphocytes/gated monocytes) determined by flow cytometry and the CD3-to-CD14 (CD3/CD14) relative protein ratios calculated from the Western blot analysis were highly correlated (r = 0.92, p < 0.001, n = 8, Fig. 5B). A positive association of CD3/CD14 to mononuclear cell LC3BII/I content was found (r = 0.76, p = 0.03, n = 8, data not shown). However, only the amount of CD14 determined by Western blot was significantly negatively associated with mononuclear cell LC3BII/I (r = − 0.63, p = 0.048, n = 8, Fig. 5C), but not the amount of CD3 (Fig. 5D). p62 protein expression was not related to CD3/CD14 relative protein ratios (r = 0.02, p = 0.96, n = 8). In addition, LC3B^+^ mononuclear cells/µl were significantly negatively related to mononuclear cell LC3BII/I (r = − 0.74, p = 0.02, n = 8, Fig. 5E), but not p62, which was due to monocytes/µl (r = − 0.77, p = 0.01, n = 8, data not shown), but not lymphocytes/µl (r = − 0.55, p = 0.10, n = 8, data not shown). Also, a negative trend was detected for the relationship between the number of Annexin V + mononuclear cells/µl and Bcl-2 in the fasting condition (rho = − 0.60, p = 0.06, n = 8, Fig. 5F).

## Discussion

This is the first study to report that within the heterogeneous group of mononuclear cells, lymphocytes were less sensitive to apoptosis and autophagy than monocytes during fasting and exercise conditions, although circulating apoptotic and autophagic lymphocyte concentrations increased after exercise, while monocytes did not. Fasting increased circulating autophagic monocyte concentrations, but not lymphocytes. Interestingly, when autophagy measured by LC3BII/I was increased during fasting, a fewer number of circulating cells were autophagic within the mononuclear cell population, which could be attributed to the monocyte subgroup. The lymphocytes/monocytes ratios measured by flow cytometry were validated by Western blotting, because we found that the CD3/CD14 ratios in the mononuclear cell protein extracts were strongly correlated with the gated cell number ratios. Also, increased autophagy was concomitantly detected with increased apoptosis by flow cytometry and Western blot for total mononuclear cells.

Among the different types of self-consumption, autophagy plays an important role in regulating apoptosis, especially after stress-inducing interventions such as fasting [18] or acute exercise [19]. It has recently been demonstrated as a critical molecular process in promoting cell survival against apoptosis [20]. Within the mononuclear cell population in a fed condition, monocytes are on average not only more affected by apoptosis than lymphocytes (63.1 vs. 2.4%, respectively, compare [9], but also by autophagy (16.2% vs. 0.13%, respectively). In general, autophagy contributes to a healthy balance of lymphocyte survival and homeostasis [21] and is essential for monocyte-macrophage differentiation [22]. Monocytes were also shown to be more autophagic than lymphocytes in healthy control participants [23] and in patients with systemic diseases such as lupus erythematosus or active tuberculosis [24, 25]. The direct comparison of the amount of autophagy-induction between cell subtypes during different stress interventions in the healthy, however, has not yet been done. This information would be important, especially when thinking of fasting and exercise as possible health promoting interventions.

Interestingly, our flow cytometry data showed that also during fasting the more mononuclear cells were apoptotic, the more were also autophagic at the same time and apoptosis was on average higher than autophagy (12.3% vs. 4.8%, respectively). Split by mononuclear cell subgroup, lymphocytes were almost not affected by the two types of self-consumption (apoptosis: 1.6% vs. autophagy: 0.27%), while monocytes showed higher values (apoptosis: 69.2% vs. autophagy: 26.4%). One would have anticipated a fasting-induced cytoprotective mechanism of autophagy [2], but comparing percentages between the two types of self-consumption, it rather seemed that fasting-induced early and mid-phase apoptosis overcame autophagy, both in lymphocytes and monocytes. The high amount of apoptosis could have resulted in the subversion of cytoprotective mechanisms, including autophagy [2]. Fasting has also been hypothesized as a priming tool against viral infection based on its stimulating effect on autophagy in immune cells [26]. Recently, fasting reduced the number of circulating pro-inflammatory monocytes due to cell accumulation in the bone marrow [27] and the loss of a large portion of immune cells in the gut was due to apoptosis [28]. Our intervention of 14–15 h time-restricted fasting did not affect absolute circulating numbers of either lymphocytes or monocytes and enhanced only monocyte autophagy compared to the fed condition—possibly a reaction to make monocytes less inflammatory [27]. The amount of autophagy can vary between cells, even within a homogenous cell population [7]. Our results indicate a negative relationship between the amount of autophagy measured by LC3BII/I and circulating autophagic mononuclear cell numbers, which could indicate more autophagy per single mononuclear cell, possibly representing a general mechanism for context-specific regulation of cell fate by autophagy [7]. This association could not be reproduced in the lymphocyte, but only in the monocyte subgroup, both by flow cytometry gating for LC3B + cell subgroups and Western blot analyses for CD3, CD14, and LC3BII/I. This validated that the amount of autophagy was linked to the amount of monocytes present, but still did not answer if the amount of autophagy per single monocyte depended on the circulating number of autophagic monocytes. Therefore, further analyses in only the monocyte subgroup—possibly by investigating the amount of autophagy within a single monocyte using different monocyte concentrations—are warranted.

Importantly, we validated the lymphocyte-to-monocyte ratios analyzed by flow cytometry by the CD3-to-CD14 ratios detected by Western blot in the fasting condition. The strong association between the two different methodologies reassured that we were analyzing similar cellular compositions. We also showed a negative relationship between the number of Annexin V + or LC3B + mononuclear cells/µl analyzed by flow cytometry and mononuclear cell LC3BII/I or Bcl-2 assessed by Western blot in the fasting condition, respectively. These results suggest a link between the two different methodologies.

Acute exercise promotes the immediate mobilization and redistribution of effector lymphocytes to e.g. the upper respiratory tract aiming at fighting pathogens and enhancing immune surveillance [29]. Considering the normally occurring lymphocytosis post-acute exercise, as also seen in our study, it is interesting that on average only 0.11% of circulating lymphocytes were autophagic, while 11.8% of monocytes showed autophagy-induction. Especially when considering that the increase in lymphocyte concentrations post-exercise favors the Th1-mediated immune response, protecting against infections by intracellular microorganisms [29]. A similar picture arises when looking at post-exercise apoptosis, where only 1.7% of lymphocytes and 50.3% of monocytes are apoptotic. The necessity of autophagy and apoptosis for an acute exercise-induced immune response should be further investigated, especially considering cellular protection against viral infection by different T-cell subgroups.

A limitation of the current study is the low number of participants and the fact that only 5 out of 8 participants were willing to do the exhaustive exercise test. Furthermore, our Western blotting analyses do not directly show from which mononuclear cell subgroup the Bcl-2, LC3B, and p62 signals are derived. Ideally, one would have first sorted the subpopulations of living mononuclear cells into lymphocytes and monocytes and then analyzed them separately. Nevertheless, our correlation analysis suggests an association between the LC3B signal and the monocyte subgroup. To better understand the distinct behaviors of LC3BII/I and p62 and to further characterize the effect of fasting and exercise on autophagy flux, samples of additional participants would need to be analyzed in the absence and presence of autophagy inhibitors.

## Conclusion

Flow cytometry identified monocytes as being more apoptotic and autophagic than lymphocytes during fasting and exercise conditions. Both types of self-consumption are essential for immune adaptations to certain stress situations. This underlines the importance of fasting and exercise for the stimulation of apoptosis and autophagy in pro-inflammatory monocytes, as this cell-type may be instrumental in the health-promoting effects of these interventions.

## Data Availability

All data generated or analyzed during this study are included in this published article.
